# Exploring the Revolutionary Impact of YAP Pathways on Physical and Rehabilitation Medicine

**DOI:** 10.3390/biom15010096

**Published:** 2025-01-10

**Authors:** Carmelo Pirri

**Affiliations:** Department of Neuroscience, Institute of Human Anatomy, University of Padova, 35121 Padova, Italy; carmelop87@hotmail.it or carmelo.pirri@unipd.it

**Keywords:** YAP, Hippo, TAZ, exercise, sport, rehabilitation, physical therapy, muscle, bone, cartilage

## Abstract

Cellular behavior is strongly influenced by mechanical signals in the surrounding microenvironment, along with external factors such as temperature fluctuations, changes in blood flow, and muscle activity, etc. These factors are key in shaping cellular states and can contribute to the development of various diseases. In the realm of rehabilitation physical therapies, therapeutic exercise and manual treatments, etc., are frequently employed, not just for pain relief but also to support recovery from diverse health conditions. However, the detailed molecular pathways through which these therapies interact with tissues and influence gene expression are not yet fully understood. The identification of YAP has been instrumental in closing this knowledge gap. YAP is known for its capacity to perceive and translate mechanical signals into specific transcriptional programs within cells. This insight has opened up new perspectives on how physical and rehabilitation medicine may exert its beneficial effects. The review investigates the involvement of the Hippo/YAP signaling pathway in various diseases and considers how different rehabilitation techniques leverage this pathway to aid in healing. Additionally, it examines the therapeutic potential of modulating the Hippo/YAP pathway within the context of rehabilitation, while also addressing the challenges and controversies that surround its use in physical and rehabilitation medicine.

## 1. Introduction

Physical and rehabilitation medicine, including physical therapies, exercise, ultrasound, vibration, cold therapy, hyperthermia, and manual therapy, etc., employs mechanical and thermal stimuli to achieve therapeutic effects, but the underlying mechanism of these interventions is incompletely understood. The Hippo/YAP signaling pathway provides a new perspective on how physical therapies contribute to tissue repair and regeneration. YAP, as a key effector of this pathway, acts as a sensor of mechanical cues such as extracellular matrix stiffness, fascial stiffness, blood flow, and muscle contractions, converting these inputs into cellular responses that affect proliferation, differentiation, and migration. The Hippo/YAP signaling pathway was initially identified in Drosophila, where it plays a fundamental role in controlling organ size by regulating cell proliferation and apoptosis [1]. In mammals, this pathway has been shown to be highly conserved, with Yes-associated protein (YAP) and transcriptional coactivator with PDZ-binding motif (TAZ) serving as the primary effectors that translocate to the nucleus in response to changes in mechanical cues and promote the transcription of targeted genes [2]. The Hippo/YAP pathway is regulated through a kinase cascade that includes mammalian STE20-like protein kinases 1 and 2 (MST1/2) and large tumor suppressor kinases 1 and 2 (LATS1/2), which phosphorylate YAP/TAZ and prevent their nuclear localization [3].

The application of mechanical stimuli, such as those encountered during physical therapy, is known to influence cellular fate and tissue homeostasis [4,5]. These stimuli, including therapeutic exercise, ultrasound, manual therapy, physical therapies, and mechanical loading, have been shown to affect processes such as stem cell differentiation, tissue remodeling, and inflammatory response [6]. For instance, mechanical forces generated during exercise or ultrasound therapy have been demonstrated to influence YAP activity and promote tissue regeneration and repair [4,5,7]. Recent studies have further demonstrated that mechanical stimuli, including vibration therapy and mechanical stretching, can enhance YAP-mediated signaling, thereby promoting angiogenesis and muscle regeneration [8]. This mechano-transduction capability makes the Hippo/YAP pathway a key player in rehabilitation medicine, where mechanical signals are harnessed to stimulate tissue repair and remodeling, reduce inflammation, and improve functional recovery. Furthermore, physical therapies are particularly relevant in the context of musculoskeletal, cardiovascular, and metabolic diseases, where tissue regeneration and remodeling are crucial components of rehabilitation [4]. The integration of the Hippo/YAP pathway in these therapies provides a molecular understanding of how mechanical and thermal signals are translated into beneficial biological outcomes to enhance the effectiveness of rehabilitation strategies. Understanding the specific role of YAP in different therapeutic modalities will aid in optimizing treatment protocols and potentially combining various forms of physical therapies, exercise, and manual therapy, etc., for enhanced outcomes [9].

The aim of this narrative review is to explore the role of the Hippo/YAP pathway in physical and rehabilitation medicine, delving into how this mechanism can explain the therapeutic effects of the different therapies of physical and rehabilitation medicine. Specifically, we will focus on how the mechanical and thermal stimuli used in these therapies—such as physical exercise, ultrasound, vibration, manual therapy, and cryotherapy—influence YAP activation, and consequently the cellular processes involved in tissue repair and regeneration. A deeper understanding of how YAP responds to mechanical stimuli will allow for the identification of new strategies to optimize the effectiveness of rehabilitative therapies, potentially combining different modalities to maximize regenerative effects and reduce inflammation. This integrated approach could lead to significant improvements in the management of musculoskeletal, cardiovascular, and metabolic conditions, enhancing functional recovery and the patient’s quality of life.

## 2. Generality on Yes-Associated Protein (YAP)

**Figure 1:**
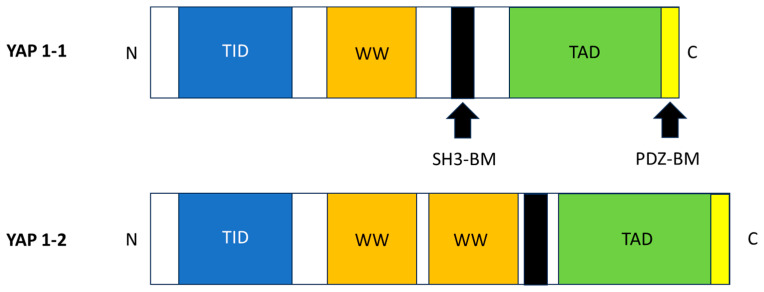
Modular structure of YAP.

Yes-associated protein (YAP) is a pivotal effector of the Hippo signaling pathway, a highly conserved kinase cascade that plays a critical role in regulating fundamental cellular processes such as proliferation, differentiation, and apoptosis [1]. YAP1 was first identified and named by Sudol et al. in 1995, who discovered and cloned this novel 65 KD protein. It was found to bind to the SH3 domain of the non-receptor tyrosine kinase YES [10]. The gene product of YAP1 has two main subtypes, YAP1-1 and YAP1-2, which differ by an extra 38 amino acids that form the WW domain. The YAP1-1 variant, containing a single proline, is also known as Yes-associated protein (YAP65) due to its binding to the proto-oncogene YES. In mammals, the YAP1 ortholog has either one (YAP1-1) or two (YAP1-2) WW domains. Structurally, YAP1 includes a transcription activator-binding protein (TID), a WW domain, and a transcription activation domain (TAD). Some YAP1 variants also feature an SH3-binding motif (SH3-BM) and a leucine zinc finger. At its N-terminal, YAP1 has a proline-rich region, followed by the TAD and PDZ-binding motif (PDZ-BM), which are crucial for activating specific gene transcription. Due to regulatory variations in transcription, translation, and modification levels, YAP1 functions differently across cell types. The modular structure of the two YAP1s is illustrated in Figure 1.

**Figure 2:**
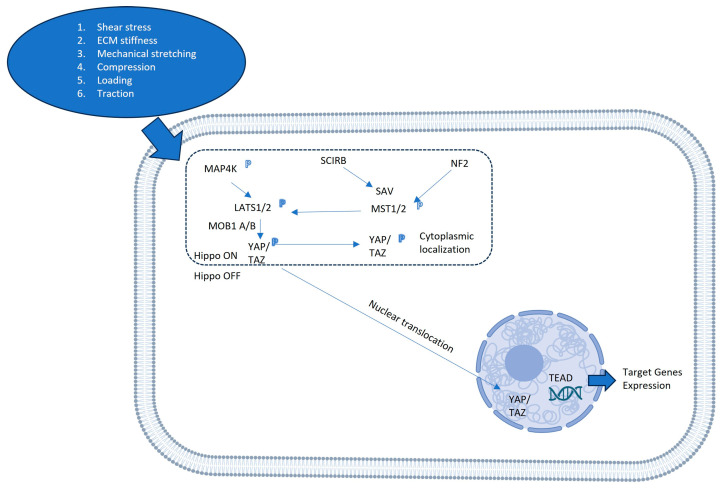
Mechano-transduction pathways regulating YAP/TAZ activity. This schematic shows how mechanical signals—such as ECM stiffness, mechanical stretch, and shear stress—modulate YAP/TAZ activity via Hippo-dependent and independent pathways. The Hippo pathway’s core kinases, MST1/2 and LATS1/2, respond to mechanical cues to regulate YAP/TAZ phosphorylation and localization. Alternatively, these mechanical stimuli can bypass the kinase cascade, influencing YAP/TAZ localization through cytoplasmic and nuclear actin dynamics.

The Hippo signaling pathway acts as a central regulator of organ size, tissue homeostasis, and regeneration, with YAP being one of the primary mediators through which these effects are exerted. Together with its homolog, transcriptional coactivator with PDZ-binding motif (TAZ), YAP functions as a mechanosensitive transcriptional regulator that integrates a variety of extracellular signals—including mechanical forces, oxidative stress, and biochemical cues—into specific cellular responses that determine cell fate [4,9]. The Hippo pathway itself consists of a series of kinases, including MST1/2 and LATS1/2, which phosphorylate and inactivate YAP/TAZ, thus retaining them in the cytoplasm [10]. When the Hippo pathway is inactive, YAP and TAZ translocate to the nucleus, where they interact with transcriptional enhancer activator domain (TEAD) family proteins, among others, to drive the transcription of genes involved in cell growth, survival, and tissue regeneration [11]. The activity of YAP is tightly regulated by upstream signals, such as cell–cell contact, cytoskeletal dynamics, and extracellular matrix (ECM) stiffness, allowing YAP to act as a key sensor of the mechanical and biochemical state of the tissue environment [3]. Mechanical stress is one of the most critical regulators of YAP activity. Cells residing within tissues are constantly exposed to mechanical forces, such as tension, compression, and shear stress, which alter the physical properties of the ECM. YAP/TAZ serve as mechano-transducers, responding to these forces by modulating gene expression programs that control cell proliferation and differentiation [4]. When cells experience high mechanical tension or increased ECM stiffness, YAP becomes activated and accumulates in the nucleus, promoting the expression of genes that drive cell growth and survival [5]. Conversely, under conditions of low mechanical tension, YAP is phosphorylated and sequestered in the cytoplasm, leading to decreased cell proliferation and increased differentiation and apoptosis. This mechanosensitive function of YAP is important in maintaining tissue homeostasis and orchestrating appropriate responses to injury and regeneration (Figure 2) [12].

YAP is also highly responsive to biochemical signals, including growth factors, hormones, and oxidative stress. Growth factors such as the epidermal growth factor (EGF) and transforming growth factor-β (TGF-β) can activate YAP through various signaling pathways, including the PI3K-Akt and Rho GTPase pathway, enhancing its nuclear localization and transcriptional activity [10,13]. YAP’s interaction with these pathways allows it to act as a central integrator of diverse signaling inputs, thereby playing a key role in coordinating complex cellular responses, such as stem cell maintenance, tissue repair, and immune modulation [14]. Oxidative stress, which often occurs during inflammation or tissue damage, can also influence YAP activity, by altering its phosphorylation state or its interactions with other proteins in the cell [15]. In the nucleus, YAP functions as a transcriptional coactivator, lacking its own DNA-binding domain but instead partnering with TEAD transcription factors to regulate target gene expression [13]. The YAP-TEAD complex controls the expression of numerous genes involved in cell proliferation, such as CTGF (connective tissue growth factor) and CYR61 (cysteine-rich angiogenic inducer 61), which are crucial for tissue regeneration and wound healing [11]. Additionally, YAP interacts with other transcription factors, including SMADs, p73, and RUNX, enabling it to participate in multiple cellular pathways and processes. For example, YAP’s interaction with SMADs allows it to modulate TGF-β signaling, which is essential for fibrosis, while its interaction with RUNX2 is important for osteoblast differentiation and bone formation [16].

The regulation of YAP is also influenced by cell polarity and cell density. In conditions of high cell density, the Hippo pathway is activated, leading to YAP phosphorylation and cytoplasmic retention and inhibiting cell proliferation [13]. This contact inhibition mechanism prevents overgrowth and maintains tissue architecture. In contrast, in low-density environments or when cell polarity is disrupted, Hippo signaling is suppressed, allowing YAP to translocate to the nucleus and promote cell proliferation. This dynamic regulation underscores YAP’s role in maintaining tissue homeostasis and its potential contribution to pathological conditions when dysregulated [17]. In summary, YAP is a versatile and dynamic effector of the Hippo signaling pathway, integrating mechanical, biochemical, and environmental signals to regulate cell behavior. Its ability to modulate gene expression in response to changes in the cellular microenvironment makes YAP a critical regulator of tissue homeostasis, regeneration, and development. Given its role in mediating key processes such as cell proliferation, differentiation and apoptosis, YAP has emerged as a promising therapeutic target for various conditions, including cancer, fibrosis, and degenerative diseases [9,18].

## 3. YAP and Diseases

The Hippo/YAP signaling pathway is an essential mechano-transducer that mediates cellular responses to mechanical cues, influencing disease progression across diverse contexts, including cancer, tissue regeneration, musculoskeletal integrity, and vascular health. In cancer, YAP responds to mechanical alterations in the tumor microenvironment, increasing ECM stiffness, which has been identified as a key factor in promoting tumor proliferation and invasiveness, notably in breast cancer [19,20]. YAP activation drives the transcription of genes like Skp2, which are critical for cell cycle progression, thereby enhancing tumorigenesis [19,20]. YAP inactivation leads to cell cycle arrest, effectively curbing tumor growth [19,20]. Additionally, the accumulation of advanced glycation end-products (AGEs) in the ECM can alter its viscoelasticity, promoting oncogenic β-catenin signaling in hepatocellular carcinoma (HCC). Mechanistically, AGEs increase ECM stiffness, activating the integrin-β1–tensin-1–YAP pathway, which in turn stimulates HCC cell proliferation and invasion, underscoring YAP’s central role in converting ECM mechanical signals into tumor-promoting biochemical responses [21,22]. Beyond oncology, YAP is also essential in tissue regeneration, particularly in organs with a high regenerative capacity, like the liver. In response to mechanical cues such as shear stress, YAP facilitates hepatocyte proliferation by enabling them to re-enter the cell cycle in a process that is further amplified by integrin β1, a key shear sensor at the cell membrane [23,24]. Upon activation, YAP translocates to the nucleus, initiating transcriptional programs crucial for cell proliferation and tissue repair. Studies have shown that YAP activation following mechanical stress also supports skin regeneration by activating Engrailed-1, a protein essential for wound healing [25]. When YAP signaling is blocked, Engrailed-1 activation is inhibited, which compromises the regenerative response, highlighting YAP’s critical function in tissue repair [25]. In the musculoskeletal system, YAP mediates responses to mechanical stress in cartilage and bone, which are highly responsive to mechanical signals due to their load-bearing roles. In cartilage, YAP activation promotes chondrocyte proliferation by upregulating cell cycle proteins like PCNA and cyclin D1 [26]. The nuclear translocation of YAP also depends on the substrate stiffness; soft substrates promote the expression of cartilage-specific markers like Col2α1, preserving the chondrocyte phenotype, while stiffer substrates drive YAP nuclear localization, leading to dedifferentiation. In bone tissue, YAP contributes to osteogenic differentiation in response to mechanical loading via the Piezo1 pathway, which regulates glutaminolysis through GLS1 activity. This pathway promotes bone cell differentiation and tissue integrity by enabling cells to adapt to mechanical stress, with YAP’s role in skeletal development underscoring its importance for musculoskeletal health [27]. In vascular homeostasis, YAP’s mechano-sensitivity also impacts endothelial cell behavior, particularly in the regulation of atherosclerosis [28]. Endothelial cells are continually exposed to shear stress from blood flow, with a stable, laminar flow inhibiting YAP’s nuclear translocation, thereby reducing inflammation and oxidative stress. Disturbed or oscillatory flow, on the other hand, activates YAP, promoting atherogenic processes [29]. Mechanistically, YAP activation under a disturbed flow is regulated by integrin-Gα13 and JNK signaling pathways, while in vivo studies indicate that YAP knockdown in endothelial cells reduces plaque formation, highlighting its role in atherosclerosis progression [30]. Additionally, oscillatory shear stress induces YAP nuclear translocation, leading to endothelial activation through pathways dependent on integrin α5β1 and its downstream kinase c-Abl. Blocking these interactions reduces YAP activation and subsequently decreases early atherosclerotic changes [31]. Overall, YAP has an advanced mechanosensory function, converting physical stimuli into cellular responses that impact cell fate, growth, and disease progression. These pathways’ ability to integrate and transduce complex mechanical signals positions YAP as a critical target for innovative treatments aimed at altering mechanical dynamics within tissues to modulate disease outcomes.

## 4. Hippo/YAP Signaling Pathway in Musculoskeletal Rehabilitation

**Figure 3:**
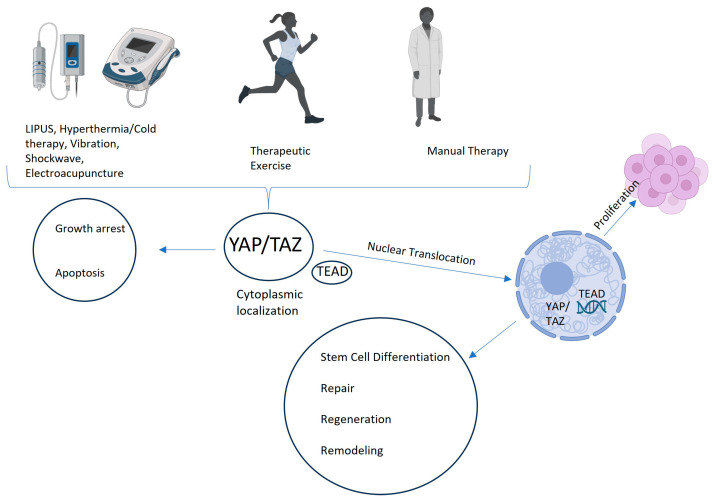
Hippo/YAP signaling pathway in physical and rehabilitation medicine. The Hippo/YAP pathway responds to mechanical stimuli, promoting tissue repair and regeneration in cartilage, bone, muscle, blood vessels, nerves, fascia, and tendons. YAP activation under different forms of mechanical loading—such as exercise, LIPUS, vibration therapy, etc.—reduces inflammation and supports cell proliferation and differentiation, as well as the regeneration and remodeling of various tissues. This pathway modulates key cellular processes, making it essential for tailoring rehabilitation protocols to enhance recovery in musculoskeletal injuries and degenerative conditions.

In musculoskeletal rehabilitation, the mechanical stress generated during exercise has been shown to regulate, for example, chondrocyte proliferation and differentiation via YAP activation. Increased YAP expression promotes chondrocyte dedifferentiation under high mechanical loads, while its inhibition preserves the differentiated chondrocyte phenotype, which is essential for cartilage health. Additionally, mechanical loading through resistance exercises has been shown to enhance osteogenic differentiation by activating YAP, which in turn increases the expression of osteogenic markers such as RUNX2 and osteopontin [32]. This suggests that the Hippo/YAP pathway not only plays a crucial role in maintaining cartilage health, but also contributes to bone regeneration and strengthening during rehabilitation. Similarly, low-intensity pulsed ultrasound (LIPUS), a common therapeutic modality, enhances tissue regeneration by promoting YAP activation, which in turn regulates the transcription of genes involved in cell proliferation and tissue repair. LIPUS has also been reported to promote muscle regeneration by enhancing satellite cell activation through the Hippo/YAP pathway [33]. This effect is beneficial for muscle recovery following injury, where promoting the proliferation and differentiation of satellite cells is critical for restoring muscle function. Moreover, vibration therapy, which delivers mechanical stimulation at specific frequencies, has been demonstrated to enhance muscle hypertrophy and bone density by regulating YAP/TAZ signaling [34]. The vibration-induced activation of YAP leads to increased muscle protein synthesis and bone mineralization, which is essential for improving musculoskeletal health, especially in older adults or individuals with reduced mobility [34,35]. Recent findings also indicate that mechanical stretching applied to tendons and ligaments can modulate YAP activity, promoting tenocyte proliferation and enhancing tendon repair [36]. The Hippo/YAP pathway also plays a role in reducing inflammation during musculoskeletal rehabilitation. Mechanical loading has been shown to inhibit pro-inflammatory cytokine production by modulating YAP activity, thereby reducing tissue inflammation and promoting an environment conductive to healing [37]. This anti-inflammatory effect of YAP-mediated signaling is important in conditions such as osteoarthritis, where chronic inflammation impairs tissue regeneration and function. Recent research has also linked YAP to macrophage polarization, indicating that high YAP expression can promote macrophage polarization towards the pro-inflammatory M1 phenotype while inhibiting the anti-inflammatory M2 phenotype. This interplay can influence inflammation and tissue repair processes during musculoskeletal rehabilitation [37]. Overall, the Hippo/YAP signaling pathway is emerging as a key regulator of musculoskeletal health, responding to mechanical stimuli to drive tissue regeneration, reduce inflammation, and promote functional recovery. By understanding the mechanisms by which YAP modulates chondrocyte, muscle, bone and fascia cell behavior, rehabilitation protocols can be better tailored to enhance patient outcomes, in particular in individuals with musculoskeletal injuries or degenerative diseases (Figure 3 and Table 1).

### 4.1. LIPUS and YAP

Low-intensity pulse ultrasound (LIPUS) is an increasingly popular therapeutic modality in regenerative medicine due to its ability to deliver mechanical stimulation in a non-invasive manner [42]. YAP is highly responsive to the mechanical cues generated by LIPUS, which can influence cellular processes [43]. Recent studies have shown that LIPUS enhances YAP nuclear translocation, thereby promoting the transcription of genes associated with cell proliferation and tissue regeneration, such as AREG, CYR61, and cyclin D1 [38,44]. In endothelial cells, LIPUS facilitates vascular remodeling by increasing YAP activity, leading to improved endothelial cell connections and angiogenesis, which is crucial for wound healing and tissue repair [45]. Additionally, LIPUS has been shown to inhibit adipocyte differentiation by enhancing YAP’s nuclear localization, resulting in decreased expression of pro-adipogenic factors and promoting a phenotype conducive to tissue regeneration [45]. These findings underscore the role of LIPUS as a promising intervention that leverages YAP’s mechanosensitive properties to enhance tissue healing and repair, making it a valuable tool in the field of regeneration medicine.

### 4.2. Vibration and YAP

Vibration therapy is another physical modality that exerts mechanical stimulation to promote tissue regeneration, with YAP playing a crucial role as a mechanosensitive regulator in this context. Vibration therapy applies cyclic mechanical forces to tissues, which enhances cellular mechano-transduction pathways, including the Hippo/YAP signaling pathway. YAP activation in response to vibration therapy has been demonstrated to promote muscle regeneration by stimulating satellite cell proliferation and differentiation, which is essential for repairing muscle damage and improving muscle strength [35,52]. Moreover, vibration-induced mechanical cues enhance osteogenic differentiation through YAP activation, promoting increased bone mineral density and bone strength, which is beneficial for individuals suffering from osteoporosis or reduced mobility [39]. Furthermore, studies have shown that vibration therapy also activates YAP/TAZ signaling in tenocytes, promoting tendon repair and improving the biomechanical properties of tendons, which is crucial for functional recovery after injury [53]. These findings highlight the potential of vibration therapy as a non-invasive intervention that harnesses YAP’s mechanosensitive properties to enhance musculoskeletal health, making it an effective therapeutic option for improving tissue repair and functional outcomes in rehabilitation.

### 4.3. Electroacupuncture and YAP

Electroacupuncture (EA), a modality combining traditional acupuncture with electrical stimulation, has emerged as a promising technique in promoting tissue repair, partly by modulating the Hippo/YAP signaling pathway. Recent studies have demonstrated that EA promotes YAP activation and nuclear translocation, thereby enhancing the expression of genes related to tissue regeneration, including CTGF and ANKRD1 [37]. In musculoskeletal injuries, EA-induced YAP activation has been found to promote the proliferation and differentiation of mesenchymal stem cells (MSCs), thereby accelerating muscle repair and bone regeneration [57]. Additionally, EA has been shown to improve the repair of peripheral nerve injuries by activating YAP/TAZ signaling, which enhances Schwann cell proliferation and supports axon regrowth [58]. In ischemic stroke models, EA has been found to upregulate YAP expression in the penumbra area—the region surrounding the infarct. This upregulation of YAP reduces apoptosis and neuroinflammation, contributing to enhanced neurological recovery. By influencing the translocation of YAP into the nucleus, EA can modulate gene expression profiles that promote cell survival, reduce oxidative stress, and improve tissue repair mechanisms [59,60,61]. Specifically, EA treatment resulted in the increased expression of mitochondrial fusion proteins such as OPA1, MFN2, and MFN1, which played critical roles in maintaining mitochondrial integrity and reducing cortical damage and apoptosis in rat models of cerebral ischemia–reperfusion injury [59,60,61]. Moreover, YAP appears to function as a key regulatory hub in this process, acting through mechano-transduction pathways to mediate cellular responses to EA-induced mechanical stimuli. The interaction between connexin 43 and YAP has also been shown to promote the nuclear translocation of YAP, leading to the activation of astrocytes and mitigating the damage caused by ischemic brain injury [59,60,61]. These findings suggest that YAP not only aids in tissue recovery by reducing apoptosis, but also modulates the inflammatory response, thereby contributing to the overall neuroprotective effects of EA. The ability of EA to stimulate the Hippo/YAP pathway highlights its potential as a therapeutic intervention for enhancing neuroplasticity and promoting recovery in neurological diseases. By targeting key molecular players involved in cell survival and regeneration, EA offers a promising approach to improving clinical outcomes in patients suffering from ischemic stroke and potentially other neurodegenerative conditions [38].

### 4.4. Hyperthermia/Cold Therapy and YAP

Hyperthermia and cold therapy are common physical therapies used to treat various rehabilitative conditions, including musculoskeletal and neurological disorders. Recent research has highlighted the involvement of Hippo/YAP signaling pathways in mediating the effects of these thermal interventions on cellular processes [46,46]. Hyperthermia, which involves the application of heat to tissues, has been shown to activate YAP by promoting its nuclear translocation. During heat stress, YAP desphosphorylates and migrates to the nucleus, where it regulates the expression of genes involved in cell survival and tissue repair. This activation of YAP enhances the heat shock response, aiding in the recovery of damaged tissue and promoting cellular regeneration [38,46,46,47]. For instance, the heat-induced activation of YAP contributes to osteogenic differentiation in ectomesenchymal stem cells, enhancing bone regeneration and repair [38,48]. Cold therapy, on the other hand, has been found to modulate YAP activity in a different manner. Exposure to cold stress increases YAP expression in certain tissues, such as the brain and adipose tissue, where it plays a crucial role in maintaining cellular homeostasis. In the context of neural development, YAP has been implicated in the cold shock response by stabilizing its expression through interactions with RNA-binding proteins like RBM3 [49]. This upregulation promotes the proliferation and differentiation of neural stem cells [50], which is essential for maintaining brain health during cold stress. Furthermore, YAP collaborates with TEAD to regulate thermogenic activity in brown adipose tissue, contributing to adaptive responses to cold exposure by promoting the expression of thermogenic genes like UCP1 [38,50,51]. These findings illustrate the dual role of YAP in the Hippo/YAP signaling pathway in response to thermal therapies, highlighting its versatility in regulating cellular adaptations to environmental changes and its potential as a target for therapeutic interventions.

### 4.5. Shock Waves and YAP

Shock wave therapy, in particular low-intensity extracorporeal shock wave therapy (LIESWT), is an emerging physical therapy that has demonstrated potential in promoting tissue repair and regeneration. The mechanical stimuli generated by shock waves have been found to impact the Hippo/YAP signaling pathway. LIESWT induces mechanical stress in targeted tissues, which subsequently activates YAP by promoting its nuclear translocation and increasing its transcriptional activity [54]. This activation of YAP plays a key role in regulating cell proliferation, differentiation, and tissue repair mechanisms [54]. In models of sciatic nerve injury, LIESWT has been shown to upregulate YAP expression and enhance its nuclear translocation, thereby promoting Schwann cell activation and facilitating nerve regeneration. The activation of YAP by LIESWT acts as an integrated transcriptional complex that modifies gene expression to support cellular repair and regeneration. Additionally, YAP has been implicated in the enhancement of angiogenesis and the recovery of injured peripheral nerves, highlighting its importance in the context of neuro-regenerative therapy. The modulation of YAP activity through shock wave-induced mechanical stimuli can provide a promising approach for enhancing tissue repair and functional recovery in various clinical applications [33,54,55]. A recent study by Pirri et al. [56] has provided further insights into the mechanobiology of YAP in response to shock wave therapy. They investigated the presence and activation of YAP in deep fascia and demonstrated that YAP activation in fascial fibroblasts increased after focal extracorporeal shock waves (fESW) treatment. This activation was associated with an upregulation of ECM components, such as type I collagen (COL1A1) and hyaluronan-binding protein 2 (HABP2), indicating that YAP is integral to the remodeling and regenerative processes of the deep/muscular fascia [56]. These findings suggest that YAP activation through mechanical cues, such as shock wave therapy, can contribute to the repair and regeneration of musculoskeletal tissues by regulating ECM composition and fascial fibroblast activity [56].

### 4.6. Exercise and YAP

Therapeutic exercise is a cornerstone of rehabilitation, involving a range of activities designed to enhance muscle strength, flexibility, endurance, and overall functional capacity. Recent research has demonstrated that during resistance training and aerobic exercises, mechanical signals generated by muscle contraction and stretching activate YAP, which translocates to the nucleus and promotes the expression of genes related to muscle growth, repair, and metabolism [40]. This process is essential for muscle hypertrophy, as well as for maintaining muscle mass during prolonged physical activity. YAP activation has also been implicated in improving mitochondrial function and metabolic regulation during endurance exercises. By enhancing mitochondrial biogenesis and promoting oxidative metabolism, YAP helps to improve muscular endurance and energy efficiency, which is beneficial in patients undergoing cardiovascular rehabilitation [41]. Moreover, studies suggest that therapeutic exercise that applies moderate to high levels of mechanical loading can enhance bone density by activating YAP in osteoblasts, promoting osteogenic differentiation and increasing bone mineral content [39]. In addition, therapeutic exercise has been shown to mitigate muscle wasting and sarcopenia through YAP-mediated pathways. In aging populations, resistance training increases YAP activity, which is associated with the activation of satellite cells and the prevention of muscle atrophy [38]. This highlights the importance of incorporating strength training exercises into rehabilitation programs for older adults, to maintain muscle mass and function [38,41]. Overall, the YAP signaling pathway is an important mediator of the adaptive responses to therapeutic exercise. By understanding how YAP contributes to muscle and bone health, as well as metabolic improvements, physiatrists can design more effective exercise programs that are tailored to the specific needs of patients, in order to optimize their recovery and functional outcomes.

### 4.7. Manual Therapy and YAP

Manual therapy, including joint mobilization, myofascial release, and spinal manipulation, etc., engages the Hippo/YAP signaling pathway to promote tissue healing and functional improvement. The mechanical forces applied during manual therapy activate YAP in a variety of cell types, including fibroblasts, chondrocytes, and intervertebral disc cells. This activation leads to several beneficial responses, such as enhanced ECM production, fibroblast proliferation, reduced apoptosis, and improved cellular resilience [40]. In particular, YAP activation has been shown to stimulate the production of key ECM components like collagen and hyaluronan, which are critical for maintaining the structural integrity of connective tissues and supporting tissue regeneration [38,40].

In intervertebral disc cells, manual therapy-induced YAP activation has been linked to reduced cell death and enhanced disc matrix synthesis, suggesting its role in mitigating degenerative disc disease. Moreover, YAP activation enhances the expression of fibrosis-related genes, including COL1A1 and HABP2 [40]. Furthermore, YAP signaling contributes to the modulation of inflammation by reducing pro-inflammatory cytokine production, thereby facilitating an environment conducive to tissue repair and pain reduction. This modulation occurs through pathways involving RhoA and ROCK, which are downstream effectors of YAP that influence cytoskeletal dynamics and cellular migration [38]. The role of YAP in mechano-transduction highlights how manual therapy can optimize tissue remodeling processes, enhance musculoskeletal function, and improve overall mobility. Understanding the molecular basis of these interventions provides valuable insight into how manual therapies can be tailored to maximize regenerative outcomes, offering a mechanistic foundation for the development of more targeted rehabilitation protocols.

## 5. Discussion

The Hippo/YAP signaling pathway plays a central role in mediating the effects of various rehabilitation interventions, spanning musculoskeletal, neurological, cardiovascular, and metabolic health. By understanding how YAP functions in response to different stimuli—including mechanical stress, thermal change, therapeutic exercise, and manual therapy, etc.—researchers and clinicians can create more targeted and effective rehabilitation protocols to promote tissue repair and functional recovery. In musculoskeletal rehabilitation, mechanical stimuli such as those generated through resistance exercise, ultrasound, vibration, and manual therapy, etc., activate YAP to regulate cell proliferation, differentiation, and tissue remodeling. The activation of YAP in chondrocytes, osteoblasts, fascial fibroblasts, and satellite cells has significant implications for cartilage health, bone regeneration, and muscle repair. For instance, YAP-mediated osteogenic differentiation in response to mechanical loading has the potential to accelerate bone healing, which is critical in patients recovering from fractures or osteoporosis. Similarly, the role of YAP in muscle hypertrophy and regeneration, in particular through satellite cell activation, underscores its importance in the rehabilitation of musculoskeletal injuries and preventing age-related sarcopenia [32,33,34,35,36,37,38,39,42,43,44,45,52,53].

Therapeutic exercise has emerged as a cornerstone of rehabilitation due to its ability to activate YAP and promote beneficial adaptations in muscle and bone tissues. By enhancing mitochondrial function, improving metabolic regulation, and driving anabolic processes, YAP activation supports the overall health and resilience of the musculoskeletal system [38,40,41]. These benefits are pronounced in older adults, where resistance training can mitigate muscle atrophy and improve functional capacity. The evidence suggests that tailoring exercise programs to optimize YAP activation can significantly enhance outcomes in patients undergoing rehabilitation for musculoskeletal disorders [38,40,41].

The role of YAP in vascular homeostasis further highlights its importance beyond musculoskeletal rehabilitation. YAP’s response to shear stress and its involvement in endothelial cell proliferation, nitric oxide production, and angiogenesis is crucial in maintaining vascular health and promoting recovery form cardiovascular conditions. YAP’s ability to regulate endothelial-to-mesenchymal transition (EndMT) also plays a pivotal role in preventing pathological fibrosis and maintaining vascular elasticity, which is essential for reducing cardiovascular risk factors and improving blood flow [38]. By modulating EndMT, YAP helps maintain endothelial cell integrity and prevents an excessive fibrotic response that can lead to vascular stiffening and reduced elasticity. Rehabilitation interventions that enhance YAP activity in endothelial cells may therefore have therapeutic potential in conditions such as atherosclerosis and peripheral artery disease. Moreover, YAP plays a role in promoting angiogenesis, which is essential for forming new blood vessels during tissue repair and in response to exercise [38]. Angiogenesis is a critical process, not only in wound healing but also in improving blood flow to ischemic tissue, which is relevant for patients with peripheral artery disease or other vascular impairments. YAP activation has been linked to the upregulation of vascular endothelial growth factor (VEGF) [38].

Thermal therapies, including hyperthermia and cold treatments, provide additional avenues for modulating YAP activity. The heat-induced activation of YAP can enhance tissue repair by promoting stem cell differentiation towards osteogenesis, while cold therapy’s ability to reduce inflammation through YAP modulation highlights its role in creating a favorable environment for healing [46,46,47,48,49,50,51]. The dynamic application of heat and cold therapies, tailored to the specific needs of the patient, can provide synergistic benefits by leveraging the YAP pathway to optimize tissue repair, reduce pain, and improve overall recovery [46,46,47,48,49,50,51].

Manual therapy, including joint mobilization, fascial manipulation, spinal manipulation, and myofascial release, etc., also engages the Hippo/YAP signaling pathway to promote tissue healing and functional improvement. Mechanical forces applied during manual therapy can activate YAP in various cell types, leading to enhanced extracellular matrix production, reduced apoptosis, and tissue remodeling. Understanding the molecular basis of manual therapy provides valuable insights into how these interventions can be optimized to promote tissue regeneration, reduce pain, and improve mobility [40].

Finally, YAP’s role in inflammation is complex, as it can act as both a pro- and anti-inflammatory mediator depending on the context [37]. In musculoskeletal rehabilitation, YAP’s influence on macrophage polarization suggests that targeting YAP could help balance inflammation and tissue regeneration, providing new opportunities for improving rehabilitation outcomes [37]. Similarly, in vascular rehabilitation, YAP’s interaction with NF-kb signaling may be leveraged to prevent vascular inflammation and enhance tissue repair in conditions such as atherosclerosis. Understanding these dual roles of YAP will be crucial for developing targeted interventions that can maximize its therapeutic benefits while minimizing potential adverse effects.

### Future Perspectives in Rehabilitation

The rehabilitative potential of YAP-mediated mechanisms represents an exciting frontier in the field of rehabilitation. While existing research has laid a strong foundation, there remain several critical areas for investigation that could significantly enhance the rehabilitative applicability of these mechanisms: (1) personalized rehabilitation protocols; (2) the integration of innovative technologies; (3) rehabilitative validation and long-term safety; (4) collaborative and interdisciplinary research; and (5) exploring synergistic effects with other rehabilitation approaches.

1. Personalized rehabilitation protocols: rehabilitation outcomes often vary widely among individuals due to a range of factors, including age, underlying comorbidities, genetic predispositions, and even the biomechanical environment of the affected tissue. Developing personalized protocols that leverage YAP activation could transform rehabilitation practices by optimizing interventions for specific patient populations. For example, the mechanical parameters of LIPUS or vibration therapy—such as intensity, frequency, and duration—could be fine-tuned based on individual biological responses. Furthermore, understanding how systemic conditions (e.g., diabetes or osteoporosis, etc.) influence YAP activation may allow for the customization of therapeutic approaches, particularly for populations with impaired healing capabilities. This personalized approach could enhance efficacy, reduce recovery times, and minimize the risk of adverse effects.
2. Integration of innovative technologies: advances in wearable technology, machine learning, and bioengineering present unparalleled opportunities to enhance the monitoring and optimization of rehabilitation strategies. Wearable devices equipped with biomechanical sensors could track real-time data in the mechanical stimuli applied during therapy and correlate these with patient-specific YAP-related outcomes. Machine learning algorithms could analyze large datasets to identify the most effective rehabilitation strategies, offering predictive insights and enabling the continuous optimization of therapy. Additionally, technologies such as 3D bioprinting and tissue-engineered scaffolds could be designed to mimic the specific mechanical properties known to activate YAP, providing new avenues for tissue regeneration and repair in challenging cases, such as chronic injuries or post-surgical recovery.
3. Clinical validation and long-term safety: while preclinical studies have demonstrated the potential of YAP-targeted interventions, translating these findings into routine clinical practice remains a challenge. Future research should prioritize well-designed, multicenter clinical trials to validate the safety, efficacy, and scalability of therapies such as LIPUS, vibration therapy, and manual therapy, etc. Special attention should be paid to identifying optimal dosing regimens and understanding the limitation of these techniques in various patient populations. Additionally, longitudinal studies are essential to investigate the long-term effects of sustained YAP activation. Questions regarding potential risks, such as fibrosis, hypertrophy, or other maladaptive responses must be addressed to ensure that these interventions are both effective and safe over extended periods.
4. Collaborative and interdisciplinary research: given the complexity of YAP-mediated mechanisms, interdisciplinary collaboration between researchers in biomechanics, molecular biology, bioengineering, and clinical rehabilitation is crucial. Such collaborations could drive the development of integrative models that account for the diverse roles of YAP in cellular adaptation, tissue repair, and functional recovery. Moreover, fostering partnerships between academia, industry, and healthcare providers may accelerate the translation of laboratory findings into practical, accessible solutions for patients.
5. Exploring the synergistic effects between different rehabilitation approaches: an exciting area of future research lies in investigating the potential synergy between YAP-targeted therapies and other established rehabilitation approaches, such as exercise-based therapy, pharmacological targets, or regenerative medicine techniques. For instance, combining mechanical therapies like LIPUS with growth factor delivery or stem cell therapies may enhance YAP activation and its downstream effects, leading to improved outcomes in tissue repair and regeneration. Similarly, understanding how YAP-mediated mechano-transduction interacts with systemic therapies for inflammation or oxidative stress could reveal new avenues for integrative treatment strategies.

By addressing these future directions, the field of rehabilitation stands poised to unlock the full potential of YAP-mediated mechanisms. The integration of personalized protocols, cutting-edge technologies, and rigorous clinical validation could not only improve patient outcomes, but also establish a new paradigm for evidence-based rehabilitation strategies.

## 6. Conclusions

YAP, with its signaling pathways, in particular Hippo/YAP, represents a pivotal mechanism through which various interventions used in physical and rehabilitation medicine exert their therapeutic effects. By acting as a mediator of mechanical stimuli, YAP plays a fundamental role in regulating tissue regeneration, reducing inflammation, and promoting functional recovery across different rehabilitation modalities. The insights gained from understanding YAP’s role in musculoskeletal health, neurological health, and vascular homeostasis offer significant opportunities for enhancing rehabilitation outcomes. Future research should continue to explore how the targeted activation or inhibition of YAP can be leveraged to maximize the benefits of physical and rehabilitation medicine, leading to more effective and personalized rehabilitation strategies for patients with diverse medical conditions.

## Figures and Tables

**Table 1:** The role of physical and rehabilitation medicine in modulating YAP. The table summarizes the intervention, the mechanism of YAP activation, YAP-mediated effects, and rehabilitative implications.

Intervention	Mechanism of YAP Activation	YAP-Mediated Effects	Rehabilitative Implications	References
Exercise (Including aerobic)	Mechanical stress and the ELABELA-APJ-Akt/YAP axis activate YAP through signaling pathway.	Enhances tissue repair by promoting cell migration and angiogenesis and reducing apoptosis.	Improves functional recovery, supports cardiovascular and musculoskeletal health, and prevents deconditioning.	[38,39,40,41]
LIPUS (Low-Intensity Pulsed Ultrasound)	Mechanical vibrations enhance YAP nuclear translocation by stabilizing the cytoskeleton and reducing phosphorylation.	Stimulates tissue regeneration, accelerates fracture healing, and inhibits adipocyte differentiation.	Optimizes recovery form fractures, supports weight management, and promotes healing in soft tissue injuries.	[38,42,43,44,45]
Cold therapy	Activates YAP via RBM3 or miR-429 pathways in response to low temperatures.	Promotes metabolic adaptation, browning of white adipose tissue, and neuroprotection.	Reduces inflammation, supports neural recovery, and aids in metabolic rehabilitation post injury or diseases.	[38,46,46,47,48,49,50,51]
Hyperthermia	Enhances YAP nuclear activity through dephosphorylation of LATS1 under thermal stress.	Promotes osteogenesis and differentiation of ectomesenchymal stem cells for tissue repair.	Facilitates recovery from bone injuries, accelerates tissue repair, and relieves musculoskeletal pain.	[38,46,46,47,48,49,50,51]
Manual therapy	Modulates Hippo/YAP activity by altering cytoskeletal dynamics through mechano-transduction.	Enhances tissue flexibility, reduces local inflammation, and improves cellular recovery in affected regions.	Restores range of motion, alleviates pain, and promotes musculoskeletal and postural recovery.	[38,40]
Vibration (Low-intensity)	Mechanical oscillations activate YAP via RhoA signaling and cytoskeletal remodeling.	Enhances mesenchymal stem cell proliferation, supports osteogenic differentiation, and maintains bone density.	Prevents bone loss, supports muscle recovery, and improves tissue integrity in elderly or immobilized patients.	[35,39,52,53]
ESWT (Extracorporeal Shock Wave Therapy)	Stimulates YAP/TAZ via ECM-cytoskeleton interactions.	Facilitates connective tissue remodeling and nerve regeneration, promotes axonal repair, and enhances Schwann cell activity.	Improves peripheral nerve recovery, accelerates functional restoration, and mitigates neuropathic deficits.	[33,54,55,56]
Electroacupuncture	Electrical and mechanical stimuli activate YAP and mitochondrial fusion processes.	Reduces neuroinflammation, promotes neuroprotection, and enhances tissue repair.	Supports functional recovery post stroke, reduces neurological deficits, and aids in chronic pain management.	[37,38,57,58,59,60,61]

## Data Availability

No new data were created or analyzed in this study.
